# Genetic Diversity, Biochemical Properties, and Detection Methods of Minor Carbapenemases in Enterobacterales

**DOI:** 10.3389/fmed.2020.616490

**Published:** 2021-01-20

**Authors:** Rémy A. Bonnin, Agnès B. Jousset, Cécile Emeraud, Saoussen Oueslati, Laurent Dortet, Thierry Naas

**Affiliations:** ^1^Team “Resist” UMR1184 “Immunology of Viral, Auto-Immune, Hematological and Bacterial diseases (IMVA-HB),” INSERM, Université Paris-Saclay, CEA, LabEx LERMIT, Faculty of Medicine, Le Kremlin-Bicêtre, France; ^2^Associated French National Reference Center for Antibiotic Resistance: Carbapenemase-Producing Enterobacteriaceae, Le Kremlin-Bicêtre, France; ^3^Evolution and Ecology of Resistance to Antibiotics Unit, Institut Pasteur-APHP-Université Paris-Sud, Paris, France; ^4^Bacteriology-Hygiene Unit, Assistance Publique-Hôpitaux de Paris, AP-HP Paris Saclay, Bicêtre Hospital, Le Kremlin-Bicêtre, France

**Keywords:** CHDL, carbapenem, transposon, insertion sequence, antibiotic resistance, detection

## Abstract

Gram-negative bacteria, especially Enterobacterales, have emerged as major players in antimicrobial resistance worldwide. Resistance may affect all major classes of anti-gram-negative agents, becoming multidrug resistant or even pan-drug resistant. Currently, β-lactamase-mediated resistance does not spare even the most powerful β-lactams (carbapenems), whose activity is challenged by carbapenemases. The dissemination of carbapenemases-encoding genes among Enterobacterales is a matter of concern, given the importance of carbapenems to treat nosocomial infections. Based on their amino acid sequences, carbapenemases are grouped into three major classes. Classes A and D use an active-site serine to catalyze hydrolysis, while class B (MBLs) require one or two zinc ions for their activity. The most important and clinically relevant carbapenemases are KPC, IMP/VIM/NDM, and OXA-48. However, several carbapenemases belonging to the different classes are less frequently detected. They correspond to class A (SME-, Nmc-A/IMI-, SFC-, GES-, BIC-like…), to class B (GIM, TMB, LMB…), class C (CMY-10 and ACT-28), and to class D (OXA-372). This review will address the genetic diversity, biochemical properties, and detection methods of minor acquired carbapenemases in Enterobacterales.

Nowadays antimicrobial resistance is of critical concern. Since the first identification of an enzyme able to destroy the penicillin by Abraham and Chain in the 1940s (1), more than 4,900 β-lactamases have been reported (http://www.bldb.eu/). Two main classifications are used: (i) the structural classification of Ambler and (ii) the functional classification of Bush and Jacoby (2, 3). Since the functional classification is more complicated, Ambler's classification will be used in this review. According to Ambler's structural classification, four classes (A to D) of β-lactamase are described (4). Briefly, the class A groups penicillinases and their extended-spectrum variants that are inhibited by clavulanate, sulbactam, and tazobactam. The class B corresponds to metallo-β-lactamases. All of the metallo-β-lactamases possess a carbapenemase activity. They are inhibited by ion chelator such as EDTA but not by class A inhibitor (clavulanate, tazobactam, sulbactam). Class C enzymes correspond to cephalosporinase and initially demonstrated better activity toward first generations cephalosporins compared to penicillins. They are inhibited by cloxacillin. Finally, class D enzymes, also named oxacillinases, group very diverse β-lactamase sub-families. They were initially reported to be inhibited *in vitro* by NaCl (5). Novel inhibitors such as avibactam, relebactam, or vaborbactam that possess inhibitory activity toward class A, C, and ± D will be described further in this review.

Thirty years of carbapenemase epidemiology demonstrated that these broad-spectrum enzymes might be split in two groups, the “Big Five” carbapenemases and the “rare” carbapenemases. The “Big Five” carbapenemases corresponds to the five main carbapenemases identified worldwide being class A KPC enzymes, metallo-β-lactamases of IMP, VIM & NDM groups, and class D OXA-48-like enzymes (4–7). The rare carbapenemases constitute a diverse group of enzymes belonging to the four classes of β-lactamases. The observed lower prevalence might be due to genetic features leading to a lower spread, or to the underdetection due to the lack of specific diagnostic tests targeting these enzymes.

Before starting this journey in carbapenemases-producing bacteria, it is important to overview the important changes in bacterial nomenclature. Indeed, with the massive use of whole genome sequencing, bacterial nomenclature has evolved rapidly during the last decade. Accordingly, this nomenclature evolution will lead to some changes in the old descriptions. Enterobacteria constitute a large and diverse group of facultative aerobic, gram negative rods. They are highly diverse regarding their biochemical properties, their pathogenicity, as well as for their ecological niches. The former order of Enterobacteriales has been reorganized recently based on the phylogenetic analysis of 1,500 protein sequences and overall genome similarity (8). The order of Enterobacterales is now composed of 7 families being *Enterobacteriaceae, Erwiniaceae, Pectobacteriaceae, Yersiniaceae, Hafniaceae, Morganellaceae*, and *Budvicaceae*. The most clinically relevant bacterial genera are part of the *Enterobacteriaceae* family, including *Escherichia/Shigella, Salmonella, Klebsiella, Citrobacter*, and *Enterobacter*. *Yersiniaceae* family contains *Yersinia* spp. and *Serratia* spp. and *Morganellaceae* contains *Morganella* spp., *Proteus* spp., and *Providencia* spp. To dive deeper in Enterobacterales classification and evolution, the case of *Enterobacter* genus is a key example. Described in the 1960s, this genus comprised more than 20 species (https://lpsn.dsmz.de/genus/enterobacter) but its classification is continuingly evolving. For instance, *Enterobacter aerogenes* (also renamed *Klebsiella mobilis* in the 1970s) has been officially reclassified as *Klebsiella aerogenes* (9). Another example of this constant evolution is the reclassification of *Enterobacter sakazakii* as *Cronobacter sakazakii* (10). Moreover, it is highly difficult, even almost impossible, to decipher the *E. cloacae* complex (ECC) using classical microbiological methods even using MALDI-TOF for that purpose (11). For all these reasons, ancient description of genus and species in this manuscript should be analyzed with the prism that they were not identified with phylogenetic analysis methods and thus can be misidentified.

## Minor Class a Carbapenemases

The main class A carbapenemases in Enterobacterales correspond to KPC-type enzymes (4, 5). Beyond this major carbapenemase family, a wide diversity of unrelated minor class A carbapenemases have emerged including IMI-, FRI-, or GES-type enzymes. To complicate the situation, some of these carbapenemases possess a peculiar phenotype that can be missed on the antibiogram. This chapter will focus on the genetic diversity and phenotypes of these rarely described class A carbapenemases.

### IMI / NMC-A

**Figure 1:**
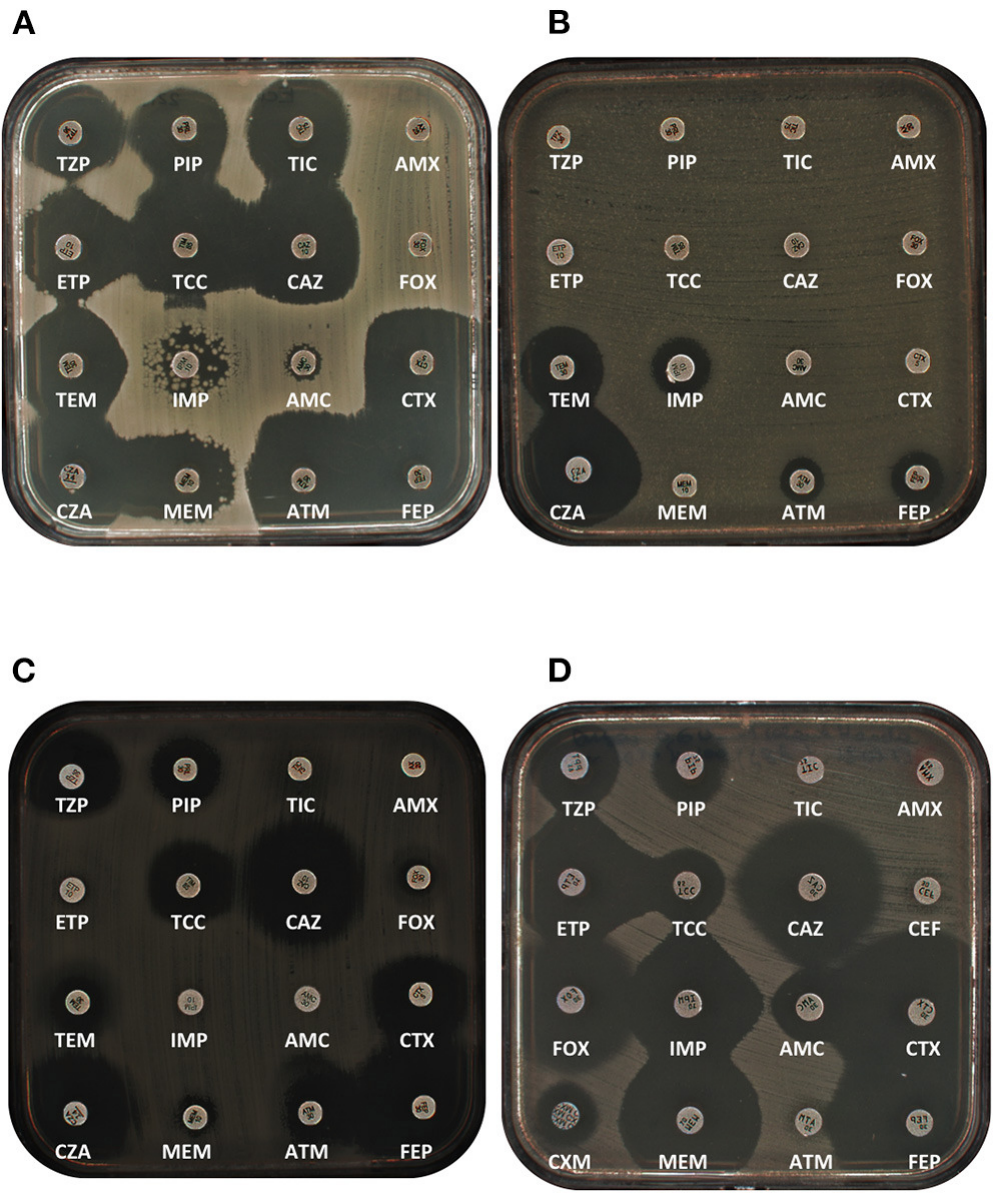
Antibiograms of representative class A carbapenemases. **(A)** IMI-1-producing *E. cloacae* complex clinical isolate. **(B)** GES-5 producing *K. pneumoniae* clinical isolate. **(C)** SME-3-producing *S. marcescens*. **(D)** FRI-1-producing *E. coli* transformant. AMX, Amoxicillin; AMC, Amoxicillin/clavulanate; ATM, aztreonam; CAZ, ceftazidime; CTX, Cefotaxime; CZA, Ceftazidime/avibactam; ETP, Ertapenem; FEP, Cefepime; FOX, Cefoxitine; IPM, Imipenem; MEM, Meropenem; PIP, Piperacillin; TCC, Ticarcillin/Clavulanate; TEM, Temocillin; TIC, Ticarcillin; TZP, Piperacillin/Tazobactam.

The IMI/NMC-A (*Imi*penemase/*N*on-*m*etallo-*c*arbapenemase A) carbapenemases form a group of carbapenemases identified in *Enterobacter* genus. They are among the oldest carbapenemases described (12, 13). IMI-1 confers resistance to penicillins alone and in combination with clavulanate, early generation cephalosporins, and carbapenems but spares broad-spectrum cephalosporins such as ceftazidime (Figure 1) (12). Despite rarely described, in comparison to “Big Five” carbapenemases, IMI-like carbapenemases have been described in different continents. IMI-1 was initially reported in the USA in an *E. cloacae* isolate from 1984, thus a year prior the US approval of imipenem. Since then, IMI-1 has been identified in *Enterobacter* genus in Singapore, China, French Polynesia, Vietnam, and Japan (14–17). IMI-2 was firstly identified in *E. asburiae* recovered from environmental samples in US rivers from 1999 to 2001 (18). It has been identified in *Klebsiella variicola* in UK, in *E. asburiae* in Czech Republic, in *E. cloacae* from Spanish rivers, France, and Canada, *Enterobacter mori* in Austria, and *E. coli* in Spain and China (19–24). IMI-3 was firstly detected in China and France (21, 25, 26). IMI-5, IMI-6 were identified in Canada (27). IMI-9 was identified in *Enterobacter cloacae* in Canada and Norway (27, 28). In addition, IMI-13 and IMI-17 were detected in France by the French National Center (unpublished data). To date, 19 variants of IMI plus NMC-A have been identified. Genetic analysis revealed interesting features related to the acquisition of those genes. Whereas, *bla*_IMI−1_ is often carried on the chromosome, *bla*_IMI−2_ is mainly carried on plasmids. The genetic element at the origin of the acquisition of *bla*_IMI−1_ is a genomic island involving XerC/XerD recombinases. These elements are named EcloIMEX-like elements (27). At least 8 EcloIMEX have been described in the literature. They differ by the diversity at the 3′ extremity of the structures. However, the 5′ extremity carrying the *bla*_IMI_-like gene is highly conserved and contained different hypothetical proteins, a putative protease, and ABC_ATPase and a glycosyltransferase (27). Some EcloIMEX elements (for XER-dependent integrative mobile elements) seem to have recombined and have lost part of their IMEX as observed for EcloIMEX-8 (17). All of these elements are inserted in a *dif* site between *setB* and *yeiP* genes. These elements are also responsible for the acquisition of *bla*_IMI−9_ carried by EcloIMEX-5 and EcloIMEX-6. The *bla*_IMI−2_ was carried on a self-transferable plasmid of ca. 66 kb in *E. asburiae* from US rivers (18). After initial identification, a *bla*_IMI−2_-carrying plasmid was sequenced from *K. variicola* (19). This plasmid belonged to IncFII-family plasmid and was of 77 kb in size. The *bla*_IMI−2_ gene has also been identified in IncFI-like plasmid in *E. coli* in Spain (20). The mechanism of acquisition of *bla*_IMI−2_ remains unclear. As for all *bla*_IMI_-like gene, a LysR family transcriptional regulator, *bla*_IMI−R_, is present upstream of *bla*_IMI−2_ (18). Different ISs or IS remnants have been identified bracketing *bla*_IMI−2_*-bla*_IMI−R_ locus including IS*Ecl3*, IS*Ecl1*, IS*Ec36* (21). The presence of this gene on IncF-type plasmid families is very likely the reason of its occurrence out of *Enterobacter* genus. The *bla*_IMI−3_ gene was identified on an IncFIIY plasmid within a new composite transposon, Tn*6306*. This transposon is composed of 2 copies of IS*Ecl1*-like bracketing the resistance gene (21). The *bla*_IMI−5_ and *bla*_IMI−6_ genes are also carried by different IncFII-type plasmids of c.a 90 and c.a 165 kb (27). Of note, the *bla*_IMI−6_ and *bla*_IMI−3_-carrying plasmids also carried a type VI secretion system that might give an advantage under certain ecological niches.

### BKC-1

BKC-1, for *B*razilian *Klebsiella c*arbapenemase, is one of the latest carbapenemases described. The first occurrence of this carbapenemase was reported in three *K. pneumoniae* strains isolated in Sáo Paulo, Brazil (29). These three isolates were recovered from two different hospitals but showed the same pulse field gel electrophoresis (PFGE) pattern and belonged to same sequence type (ST), ST1781. Cloned in *E. coli*, the production of BKC-1 confers resistance to penicillins, broad-spectrum cephalosporins, aztreonam, and decreased susceptibility to carbapenems (Table 1). However, as observed for ESBLs, efficacy of cefoxitin is not altered by BKC-1. Purification of this enzyme confirmed the observed phenotype with hydrolysis of penicillins, cephalosporins, and carbapenems but not cefoxitin. Phylogenetic analysis revealed few similarities with other class A carbapenemases, e.g., 39% of amino-acid identity with KPC-2. The closest β-lactamase corresponds to an uncharacterized β-lactamase identified in *Sinorhizobium meliloti* with 63% amino acid identity (29). The *bla*_BKC−1_ is carried by a small non-conjugative IncQ-type plasmid of 9.7 kb in size. Upstream of *bla*_BKC−1_, a copy of IS*Kpn23* is inserted, likely leading to its expression and its probable mobilization (30). Indeed, IS*Kpn23* belongs to IS*1380* family. The most famous member of this family is IS*Ecp1*, known to mobilize adjacent genes by one-ended transposition (31). This gene has also been identified in *C. freundii* harboring the same plasmid (32). Until now, this enzyme has never been described out of Brazil and presented an overall low prevalence in this country with 0.3% (2/635) of *Klebsiella* spp. isolates randomly selected among strains collected from previous surveillance studies (33). In this study, the two isolates of *K. pneumoniae* were clonally related belonging to ST442 and possessed the same plasmid.

**Table 1:** Main features of rare carbapenemases in Enterobacterales.

				**MICs of** **β-lactams in cloning vector in** ***E. coli*** **(mg/l)**						
**Name**	**Number of variants**	**Genetic environment**	**Countries of isolation**	**TIC**	**CAZ**	**IPM**	**MEM**	**ERT**	**Strain of *E. coli* and cloning vector/variant**	**References for MICs**
**Class A**										
IMI-/NMC-A	20	EcloIMEX (IMI-1-like) different ISs (IMI-2-like)	USA, Singapore, China, French Polynesia, Vietnam, Japan, Czech Republic, France, Canada, Austria, Spain, Norway	256	0.5	>32	>32	N/A	*E. coli* DH10B Vector pGB2-IMI-2	(18)
BKC	1	IS*Kpn23*	Brazil	>256	8	0.5	0.12	0.12	*E. coli* BL21 Vector pET-BKC	(29)
SHV-38	-	N/A	Brazil, India, Tunisia	>512	64	0.5	0.12	N/A	*E. coli* DH10B Vector pBK-CMV-SHV-38	(35)
CTX-M-33	-	N/A	Greece, Portugal	>512	32	1	0.25	0.25	*E. coli* TOP10 Vector natural plasmid	(42)
GES	43 (only few variants with carbapenemase activity)	integron	France, Greece, Japan, Korea, Brazil, Czech Republic, South Africa, Portugal, Belgium, Macedonia, Israel	>256	0.75	1.5	0.094	N/A	*E. coli* DH5alpha Vector PACYC184-GES-5	(47)
SFC	1	N/A	Portugal	N/A	1	4	0.38	N/A	*E. coli* XL2 pIH18	(64)
SME	5	SmarGI1-1	UK, USA, Argentina, Switzerland, Canada, Brazil	512	1	32	2	N/A	*E. coli* JM109 Vector pACYC-184-SME-1 (pTN102)	(66)
FRI	9	ISs	France, UK, Germany, Japan, Canada	>256	2	4	0.38	0.75	*E. coli* TOP10 Vector pTOPO-FRI-1	(80)
FLC	1		Netherlands	N/A	1	16	4	>2	*E. coli* LMG194 vector pBAD	(24)
**Class B**										
GIM	2	Integron	Germany	N/A	16	0.5	0.5	1	*E. coli* J53 Vector pGIM-1	(85)
KHM	1	ISs	Japan	512	>512	0.5	4	N/A		(94)
TMB	1	Integron	France	N/A	>256	1	32	N/A	*E. coli* DH5a Vector pGEM-T-TMB-1	(97)
SFH	1	N/A	Portugal	N/A	0.19	>32	>32	N/A	*E. coli* XL2 blue Vector pBGS19-SFH-1	(101)
AIM	1	IS*CR5*	China	N/A	32	0.25	0.25	1	*E. coli* TOP10 Vector pK18-AIM-1	(103)
LMB	1	ISs	Austria, Argentina	N/A	32	1	2	0.25	*E. coli* TOP10 Vector pBK-CMV-LMB-1	(108)
**Class C**										
CMY-10	-	IS*CR1*	Korea	N/A	4	0.25	0.25	N/A	*E. coli* J53 Vector natural plasmid	(111)
ACT-28	-	None	France	>256	>256	0.5	0.047	0.125	*E. coli* TOP10 Vector pTOPO-ACT-28	(110)
**Class D**										
OXA-23	-	AbaR4, Tn*6703*	France, Singapore, Finland, Belgium	>256	0.06	0.25	N/A	N/A	*E. coli* DH10B Vector pAT801-OXA-23	(196)
OXA-24	-	N/A	Algeria	>256	0.06	0.5	N/A	N/A	*E. coli* DH10B Vector pAT801-OXA-24/-40	(196)
OXA-58	-	RE elements	Belgium, Poland, Germany	>256	0.06	0.25	N/A	N/A	*E. coli* DH10B Vector pAT801-OXA-58	(196)
OXA-198	-	Integron	France	N/A	0.125	0.5	0.032	N/A	*E. coli* TOP10 Vector PUCP24-OXA-198	(138)
OXA-372	-	Tn*6256*	Italy	256	0.25	2	0.06	0.03	*E. coli* DH5alpha Vector pLBII-OXA-372	(141)
OXA-427	-	Integron	Belgium	N/A	>128	2	1	2	*E. coli* TOP10 Vector pBLII-OXA-427	(142)

*N/A: Not available*.

### SHV-38

SHV is the natural class A β-lactamase of *K. pneumoniae*. Variants of SHV with changes in their hydrolytic properties were the main resistance mechanism to broad-spectrum cephalosporins in the 1980s (with TEM-like enzyme) before the emergence and the spread of CTX-M enzymes (34). These enzymes gave the name of extended-spectrum β-lactamase compared to narrow-spectrum SHV-1. Among more than 200 variants of SHV enzymes, SHV-38, possessing A146V substitution, has been described to be the only SHV variant with carbapenemase activity (35). Once cloned in *E. coli*, this enzyme conferred increased MICs to imipenem as compared with SHV-1 (Table 1). Enzymatic assays confirmed ability of SHV-38 to hydrolyze imipenem (Table 2). However, no characterization of the impact of the amino acid substitution was performed to date (e.g., replacement of the valine by another amino acid or the crystallization of this enzyme bound to imipenem). This enzyme has been detected in Brazil, in environmental samples in India and in Tunisia, but its presence is very likely underestimated (36–38).

**Table 2:** Kinetics of minor carbapenemases.

	**Class A**										**Class B**					**Class C**			**Class D**				
**Substrates**	**Kcat (s-1)**																						
	**NMC-A**	**IMI-1**	**FRI-1**	**BKC-1**	**CTX-M-33**	**SHV-38**	**GES-5**	**SME-1**	**SFC-1**		**GIM-1**	**AIM-1**	**TMB-1**	**KHM-1**		**ACT-28**	**CMY-10**		**OXA-23**	**OXA-40**	**OXA-58**	**OXA-198**	**OXA-372**
Benzylpenicillin	260	36	1,060	34.2	210	100	317	19.3			6.6	778		23		70	3.06			5	5.5	15	40
Amoxicillin	816	190	>17,000		215	1,800		181															
Ticarcillin	81		120	1.6	7.5	10					2.3									5	1		110
Piperacillin		6.1	>26,00		205	100	4				6.9	337	3.3							1	2.5	2.6	
Oxacillin				14,306																2	1.5	25	145
Nitrocefin				22.4							5.8												
Cephalothin				118.4	380	5	49.7				16	529				384				3	0.1	0.19	0.17
Cephaloridine						40	190							686									
Cefoxitin				NH			9.6				8.3	145	0.3	1,178									
Cefotaxime	286	3.4	>220	0.4	620	1	2.9	<0.98	8.3		1.1	609		2,181		0.07				NH	NH	NH	NH
Ceftazidime	NH	<0.01	NH	0.1	0.35	110	0.3		2.1		18	7	0.07	118		0.1	5.0			20	NH	NH	NH
Cefepime			28	1.69	70	3					17	93								NH	NH	NH	NH
Aztreonam	707	51	>8,300	2.2	10	3		108	162		NH	NH	NH	NH						NH	NH	NH	NH
Imipenem	1,040	89	1,790	0.03	<0.01	0.01	1.2	104	54		27	1,700	1.7	15		0.025	1.6		0.35	0.1	0.1	0.1	5.8
Meropenem	12	10	46	0.003	0.13			8.9	6.5		2.7	1,000	1.4	0.4					0.068	NH	<0.01	0.01	0.13
Ertapenem			150	0.002	<0.01								0.4						0.021				0.49
	**Km (uM)**																						
Benzylpenicillin	28	64	567	78.7	20	13	370	16.7			46	31		1,340		36	20.5			23	50	14	110
Amoxicillin	90	780	>5,000		160	35		488															
Ticarcillin	152		393	32.7	40	14(Ki)					57									60	240		190
Piperacillin		13	>3,000		140	80	454				69	17	72							23	50	35	
Oxacillin				267.3																876	70	30	125
Nitrocefin				20.9							12												
Cephalothin					30	100	577				22	38				138				72	150	0.19	57
Cephaloridine						150	506							4.4									
Cefoxitin											206	26	69	81									
Cefotaxime	956	190	>5,000	223.9	215	800	341	NH	89		4	49		13		3.8				NH	NH	NH	NH
Ceftazidime	NH	270	NH	92.9	1,500	3,800	394	NH	52		31	148	31	8		306	33.9			2,500	NH	NH	NH
Cefepime			3,400	174.3	100	1,600					431	594								NH	NH	NH	NH
Aztreonam	125	93	>5,000	1200.7	60	5,500		259	484		NH	NH	NH							NH	NH	NH	NH
Imipenem	92	170	1,614	4.4	0.2 (Ki)	24	4.2	202	82		287	97	200	268		1.9	11.4		4.8	6.5	7.5	0.15	26
Meropenem	4.4	26	70	1.5	90			13.4	26		25	163	75	12					<1	NH	0.075	0.006	0.7
Ertapenem			98	1.7	0.009 (Ki)								31						0.5				0.25
	**Kcat/Km (uM/s)**																						
Benzylpenicillin	9.3	0.6	1.9	0.4	10	7.7	0.9	1.2			0.14	26		0.017		1.9	0.14			0.22	0.11	1.07	2.8
Amoxicillin	9.1	0.2	3.4		1.35	51		0.4															
Ticarcillin	0.5		0.3	0.05	0.2	0.7					0.04									0.02	0.004		
Piperacillin		0.5	0.9		1.5	1.3	0.009				0.1	20	0.12							0.05	0.05	0.07	0.58
Oxacillin				53.5																	0.002	0.83	1.2
Nitrocefin				1.1							0.47												
Cephalothin					12.5	0.05	0.09				0.72	14				2.8				0.05	0.001	0.02	0.003
Cephaloridine						0.27	0.4							155.9									
Cefoxitin											0.04	57	0.004	14.5									
Cefotaxime	0.3	0.02	0.04	0.002	3	0.001	0.009	NH	0.09		0.24	12		167.8		0.02				NH	NH		NH
Ceftazidime	0.05	0.00002	NH	0.001	0.0003	0.03	0.0007	NH	0.04		0.58	0.0005	0.002	14.8		0.0003	0.15			0.01	NH		NH
Cefepime			0.008	0.01	0.7	0.002					0.04	0.16								NH	NH		NH
Aztreonam	5.6	0.5	1.7	0.002	0.2	0.0005		0.4	0.004		NH	NH	NH	NH						NH	NH		NH
Imipenem	11	0.5	1.1	0.007		0.0005	0.3	0.5	0.7		0.09	17.5	0.009	0.06		0.013	0.14		0.07	0.015	0.014	0.67	0.22
Meropenem	2.7	0.4	0.7	0.002	0.0014			0.6	0.3		0.11	6,1	0.019	0.03					>0.06	NH	<0.0002	1.67	0.52
Ertapenem			1.5										0.013						0.04				0.7

*NH, Not Hydrolyzed*.

### CTX-M-33

CTX-M enzymes are the main ESBLs described worldwide and have replaced the “old” ESBLs, TEM-, and SHV-like enzymes (39). Despite the fact that the production of CTX-M-15 associated to porin deficiency can increase the MIC of ertapenem, this enzyme does not hydrolyze carbapenems at a significant level (40, 41). Recently, a variant of CTX-M-15, CTX-M-33, was reported to hydrolyze significantly carbapenems despite a very low *k*_cat_ for imipenem and ertapenem (42). This enzyme has been firstly identified in Greece in 2007 and more recently in Portugal (43, 44). As mentioned above for SHV-38, the prevalence of this enzyme is likely underestimated since sequencing of the gene is mandatory to detect this peculiar variant.

### GES

Among the wide diversity of ESBLs, some are major such as the pandemic CTX-M-family enzymes, and some are minor considering their rare identification or their restriction to certain areas. Among these ESBLs, a family is of particular interest: GES-type enzymes. Firstly identified in 1998 in France in a *K. pneumoniae* isolate (45), GES-1 production conferred resistance to penicillins, broad-spectrum cephalosporins, but not to cephamycins and carbapenems (45). After this first identification, a variant, GES-2, possessing a G170N substitution, was characterized. This variant was the first ESBL variant with a significant carbapenemase activity (46). Since then, more than 40 variants of GES-1 have been described. Among them, all variants with a substitution of the glycine 170 exhibited significant carbapenemase activity with the higher catalytic properties for G170S substitution. In addition to carbapenem hydrolysis, G170S substitution increased hydrolysis spectrum toward cephamycins (47). Noticeably, two other amino acid positions are involved in hydrolysis spectrum changes. Indeed, positions 104 and 243 are involved in increased hydrolysis toward oxyimino-cephalospoins and aztreonam, respectively (47, 48). Several GES-type carbapenemases have been identified in Enterobacterales being GES-3 in Greece, Japan, and Korea (49–51), GES-4 in Greece and Japan (49, 52), GES-5 in Korea, Brazil (including remote community in Amazonia), France (Figure 1), Czech Republic, South Africa, and Portugal (53–58), GES-6 in Belgium, Macedonia, and Israel (59, 60), and GES-16 in Brazil (61). The *bla*_GES_-type genes are usually carried by class 1 integron, but also more rarely by class 3 integron responsible for their expression. These genes have been described on a variety of plasmid families. Noticeably, this gene family is increasingly reported with, for instance, two recent reports of GES-5-producing *K. pneumoniae* in Poland and GES-5-producing *K. oxytoca, E. coli*, and *E. cloacae* in UK (62, 63).

### SFC-1

Among rare class A carbapenemases, SFC-1 (*Serratia fonticola* resistant to *c*arbapenems) and SME-like (*Serratia marcescens e*nzyme) enzymes have been identified in *Serratia fonticola* and *Serratia marcescens*, respectively (64–66). Despite its identification on the chromosome of *S. fonticola, bla*_SFC−1_ is not shared by all *S. fonticola* but only in one isolate from Portugal. No information regarding its acquisition is available in the literature. SFC-1 hydrolyzes all β-lactams including broad-spectrum cephalosporins, cephamycins, and carbapenems.

### SME

SME-1 was initially detected in two isolates recovered in England in early 1980s (67). Kinetics parameters of SME-1 and SME-2 demonstrated hydrolysis of penicillins, early-generation cephalosporins, and carbapenems but not cephamycins and broad-spectrum cephalosporins (68) (Figure 1). To date, five point-derivative variants of SME-1 were reported. SME-1 has been detected in UK and across the USA (68–71), SME-2 in Argentina, Switzerland, Canada, USA (68, 72, 73), SME-3 in USA (74), SME-4 in Brazil, Argentina, and USA (75–77), and SME-5 in Canada (Genbank accession number KJ188748). Analysis of the genetic context revealed that expression of this carbapenemase was under the control of a lysR-family transcriptional regulator, SmeR (78). SmeR acts as an inducer of carbapenemase expression in presence of cefoxitin or imipenem. Little information is available regarding the genetic environment of *bla*_SME−like_ gene. The *bla*_SME−1/−2_ genes were embedded within a 28 kb genomic island named *Smar*GI1-1. This genomic island was inserted within the chromosome of *S. marcescens* at the locus *ssrA* coding for the tmRNA representing the *att* site (79). A similar structure is responsible for the acquisition of *bla*_SME−4_ in *S. marcescens* isolate in Argentina (76).

### FRI

One of the last described class A carbapenemase family in Enterobacterales corresponds to FRI-1, for *F*rench *r*esistance to *i*mipenem (80). The *bla*_FRI−1_ gene was detected in a *E. cloacae* isolated in a patient hospitalized in Paris area, with a previous history of travel in Switzerland. Production of FRI-1 conferred resistance or reduced susceptibility to penicillins, cephalosporins, aztreonam, and carbapenems (Figure 1; Table 1). Purified FRI-1 enzyme exhibited hydrolysis of all tested β-lactams except ceftazidime. It can be noticed that *Km* were relatively high compared to other class A carbapenemases indicating a weak affinity (Table 2). Nine variants have been described to date. These variants did not correspond to point derivatives but exhibited 81–94% amino acid identities for FRI-8 and FRI-6, respectively. FRI-1 has been reported in France (80), FRI-2 in UK (81), FRI-3 in Germany (82), FRI-4 in Japan (83), FRI-5 in Japan (MH208723), FRI-6 in Canada (84), FRI-7/-8/-9 in Japan (AP019534, AP019635, AP019633). The *bla*_FRI−1_ gene was associated to a lysR-family transcriptional regulator as observed for *bla*_SME−1_ responsible for inducible expression of the gene (80). The *bla*_FRI−1_ gene has been identified on a 110 kb non conjugative and untypeable plasmid. The IncFII/IncR plasmid of 98 kb in size carrying the *bla*_FRI−4_ gene has been entirely sequenced. Interestingly, the *bla*_FRI−4_ gene and its surrounding region were duplicated on this plasmid (83). The last class A carbapenemase identified is FLC-1, for *F*RI-*l*ike *c*arbapenemase, from Indian frozen seafood in Netherland in 2017 (24). This carbapenemase is 99.66 and 82.3% amino acids identical to FRI-8 and FRI-1, respectively. Therefore, this enzyme may be reclassified as FRI-like variant. The phenotype observed in FLC-1-producing is similar to FRI-1 (Table 1). This was confirmed in biochemical analysis being hydrolysis of penicillins and carbapenems but not ceftazidime or cefepime (Table 2). The *bla*_FLC−1_ was carried by a plasmid belonging to IncFII family. Surrounding the *bla*_FLC−1_ gene, remnants of IS belonging to IS*3* family have been identified (24).

## Minor Class b Carbapenemases

Metallo-β-lactamases belong to the molecular class B of Ambler's classification and group 3 of Bush & Jacoby's classification (2, 3). These enzymes are very diverse in term of structure and hydrolytic profile. They can be classified in different subgroups (B1, B2, & B3) based on their structures. The main metallo-β-lactamases that have been identified worldwide in Enterobacterales are NDM-, VIM-, and IMP-like enzymes (4). In addition, several rare class B carbapenemases have been described in Enterobacterales being class B1 GIM-1, TMB-1, and KHM-1, class B2 SFH-1, and class B3 LMB-1 and AIM-1.

### GIM-1

**Figure 2:**
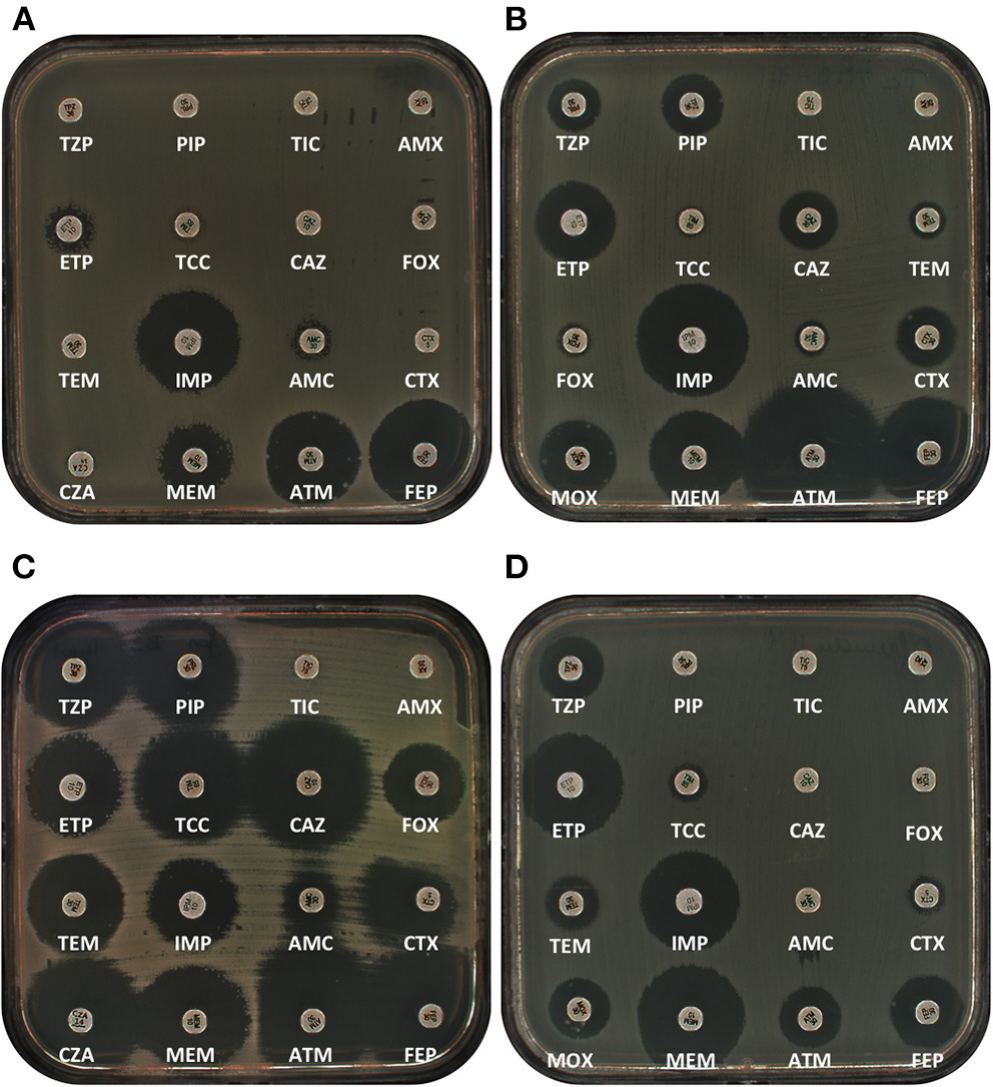
Antibiograms of representative class B carbapenemases. **(A)** GIM-1-producing *E. cloacae* complex clinical isolate. **(B)** TMB-1 producing *E. coli* transconjugant. **(C)** SFH-1-producing *S. marcescens* clinical isolate. **(D)** LMB-1-producing *C. freundii* clinical isolates. AMX, Amoxicillin; AMC, Amoxicillin/clavulanate; ATM, aztreonam; CAZ, ceftazidime; CTX, Cefotaxime; CZA, Ceftazidime/avibactam; ETP, Ertapenem; FEP, Cefepime; FOX, Cefoxitine; IPM, Imipenem; MEM, Meropenem; PIP, Piperacillin; TCC, Ticarcillin/Clavulanate; TEM, Temocillin; TIC, Ticarcillin; TZP, Piperacillin/Tazobactam.

GIM-1, for *G*ermany *Im*ipenemase, is a class B1 metallo-β-lactamase described in 2002 from five imipenem-resistant *P. aeruginosa* in Germany (85). This enzyme, as for most class B1 β-lactamases, confers resistance to penicillins, broad-spectrum cephalosporins, and carbapenems but spares aztreonam (85). It is not inhibited by clavulanate or avibactam but is inhibited *in vitro* by EDTA. Purified enzyme is able to hydrolyze all tested β-lactams except for aztreonam (Figure 2; Table 2). Crystal structures of GIM-1 revealed that the active site is narrower in comparison to VIM-1 but possessed flexibility in two loops likely explaining its wide variety of substrates (86). The *bla*_GIM−1_ gene was then identified in *S. marcescens* from a German patient over a 20-month period (87). After this first occurrence in *S. marcescens*, the *bla*_GIM−1_ gene was described in *E. cloacae, K. oxytoca, E. coli, C. amalonaticus*, and *C. freundii* (88–92). To date, this carbapenemase has spread only in Germany. Analysis of the genetic context revealed that *bla*_GIM−1_ is part of class 1 integron (85, 88, 89). This gene was carried by different conjugative plasmids but not typeable by PBRT (88, 90). Until now, only one variant, GIM-2, of this carbapenemase has been described in *E. cloacae* in Germany (93).

### KHM-1

KHM-1, for *K*yorin *H*ospital *M*etallo-β-lactamase, is a class B1 metallo-β-lactamase identified in a clinical isolate of *C. freundii* in Japan (94). This carbapenemase confers resistance to penicillins, broad-spectrum cephalosporins, and carbapenems but spares aztreonam (94). This enzyme was purified and confirmed the hydrolytic properties observed for the recombinant strain (Table 2). The *bla*_KHM−1_ gene has never been described out of Japan. However, this gene was present not only in clinical settings but also in urban sewage that might indicate a spread in the community. This carbapenemase has been identified in *C. freundii, K. quasipneumoniae*, and *E. hormaechei* subsp. *hoffmannii* (94–96). Genetic analysis revealed that *bla*_KHM−1_ is not part of class 1 integron or “classical” transposon. In *C. freundii*, genes of unknown function bracketed the *bla*_KHM−1_ (94). In *K. quasipneumoniae* and *E. homaechei*, a copy of IS*Ec68* and IS*5* have been identified, respectively (95, 96). The *bla*_KHM−1_ has been identified on different large IncA/C-type plasmids.

### TMB-1

TMB-1, for *T*ripoli *M*etallo-β-lactamase, has been described from an environmental isolate of *Achromobacter xylosoxidans* in Libyan hospital in Tripoli, Libya (97). Since then, this carbapenemase have been identified in *A. baumannii* and *Acinetobacter calcoaceticus* in Japan (98). Of note, a point derivative variant of TMB-1, TMB-2, possessing the substitution S228P, has been described in *Acinetobacter pittii* and *Acinetobacter* genomospecies 14 BJ also in Japan (99). The production of TMB-1 confers resistance to penicillins/inhibitor combinations and broad-spectrum cephalosporins but spares aztreonam, as classically observed with class B1 enzymes. Of note, MIC for meropenem is higher than for imipenem with TMB-1 (MICs at 1 mg/L vs. 32 mg/L for imipenem and meropenem, respectively). This difference was not observed with TMB-2 for which MICs to imipenem and meropenem were similar (MICs at 2 mg/L) (99). To date, only one report of TMB-1 is available in Enterobacterales from France (100). Two TMB-1-producing clinical isolates of *E. hormaechei* and *C. freundii* were recovered from a patient previously hospitalized in Tunisia (Figure 2). The *bla*_TMB−1_ gene is embedded in class 1 integron as a gene cassette always in the first position. In *A. xylosoxidans* and *Acinetobacter*, it was likely carried on the chromosome whereas it was carried by an IncN-type plasmid in Enterobacterales (100).

### SFH-1

SFH-1, for *Serratia fonticola* carbapenem *h*ydrolase, is a particular case since it corresponds to the sole characterized metallo-β-lactamase belonging to B2 subclass in Enterobacterales. This gene has been only described in *S. fonticola* in Portugal (101, 102). However, no extensive genetic analysis was performed to characterize the genetic context of this gene. Interestingly, the phenotype conferred by the production of this enzyme is peculiar. Indeed, it confers resistance to carbapenems but neither to cephalosporins nor penicilins (Figure 2; Table 1).

### AIM-1

AIM-1, for *A*delaide *i*mipene*m*ase, was described in clinical isolates of *P. aeruginosa* in Adelaide, Australia, in 2002 (103). Very few reports of AIM-1 were available in the literature. The *bla*_AIM−1_ gene has been detected in urban wastewater in West Africa and in China (104, 105). The unique description in Enterobacterales corresponds to a AIM-1-producing *K. pneumoniae* in China (104). AIM-1 producing *K. pneumoniae* was resistant to penicillins, except piperacillin, to broad-spectrum cephalosporins and carbapenems, but remained susceptible to aztreonam. Crystal structure of AIM-1 revealed that the active site is narrower than other class B3 carbapenemases (106). However, this particular conformation might explain the higher efficiency compared to other B3. The genetic context of *bla*_AIM−1_ revealed its association with IS*CR5*, an insertion sequence moving by rolling circle transposition (103). However, the acquisition mechanism remains poorly understood. In *K. pneumoniae*, no information related to the genetic context is available in the manuscript.

### LMB-1

LMB-1, for *L*inz *M*etallo-β-lactamase, is the last metallo-β-lactamase described in Enterobacterales (107). This carbapenemase has been firstly identified in *E. cloacae* in an Austrian patient hospitalized in Salzburg, Austria, in 2013. LMB-1 belongs to class B3 and the closest clinically relevant carbapenemase corresponds to AIM-1 (42% amino acid identity). However, analysis of available β-lactamase in genbank database indicated that LMB-1 presented 99% amino acid identity with a predicted β-lactamase from marine bacteria *Rheinheimera pacifica* (107). LMB-1-producing *E. cloacae* was resistant to penicillins, broad-spectrum cephalosporins, and carbapenems. Recently this carbapenemase has been identified in a clinical isolate of *C. freundii* in Buenos Aires, Argentina (Figure 2) (108). Until now, LMB-1 has not been purified due to some difficulty to express this carbapenemase (108). However, in both studies, specific activities indicated that LMB-1 hydrolyzed all β-lactams except aztreonam and cefepime. Analysis of the genetic context showed that *bla*_LMB−1_ is embedded in a complex genetic structure with different class 1 integrons. Immediately downstream of the *bla*_LMB−1_ gene, an IS*CR1* is present, whereas a putative phosphodiesterase is found upstream. This phophodiesterase is likely part of the genome of the progenitor. In both isolates, the genetic context is similar but differs in the gene cassette arrays of class 1 integrons. However, in the Austrian isolate, two copies of IS*6*-family ISs bracket the whole structure forming a putative composite transposon. The *bla*_LMB−1_ is carried on conjugative plasmids. Nevertheless, these two plasmids do not belong to same incompatibility group being IncFIb-like in Austria and IncA/C in Argentina. This is an interesting phenomenon since its identification in distant geographic areas on different plasmids might indicate that this gene has spread more than expected.

## Minor Class c Carbapenemases

Class C β-lactamases, also known as cephalosporinases, have been rarely reported as carbapenemases. To date, only two β-lactamases were described possessing a carbapenemase activity being CMY-10 and more recently ACT-28 (109, 110).

### CMY-10

CMY-10 is a point variant of CMY, the natural cephalosporinase of *Citrobacter* spp. identified in *Enterobacter aerogenes* (111). CMY-10 conferred high levels of resistance to penicillins and penicillin/inhibitor combinations and cephalosporins such as cefoxitin, cephalothin, or ceftazidime. This variant was categorized as an extended-spectrum AmpC (ESAC) due to increased MICs toward carbapenems and aztreonam (Table 1). Carbapenem hydrolysis properties were explained by a widened active site as compared to P99 AmpC due to a three amino acids deleted in R2 loop (109). The *bla*_CMY−10_ gene was carried on a conjugative plasmid associated to a complex class 1 integron (112). It is usually located at the 3' end of integron and associated with IS*CR1* leading to its expression. This β-lactamase have exclusively been reported in Korea (111–113). However, its prevalence may be underestimated. Indeed, this variant cannot be detected without sequencing and is not targeted by most detection tools available in the market. Regarding the treatment of this ESAC, scarce information is available. Avibactam is active against class C β-lactamases but it has been demonstrated that mutation in Ω-loop can increased MICs of ceftazidime-avibactam (114). Another study demonstrated that nucleotides guanosine monophosphate (GMP) and inosine monophosphate (IMP) are potential inhibitors for this enzyme (115).

### ACT-28

**Figure 3:**
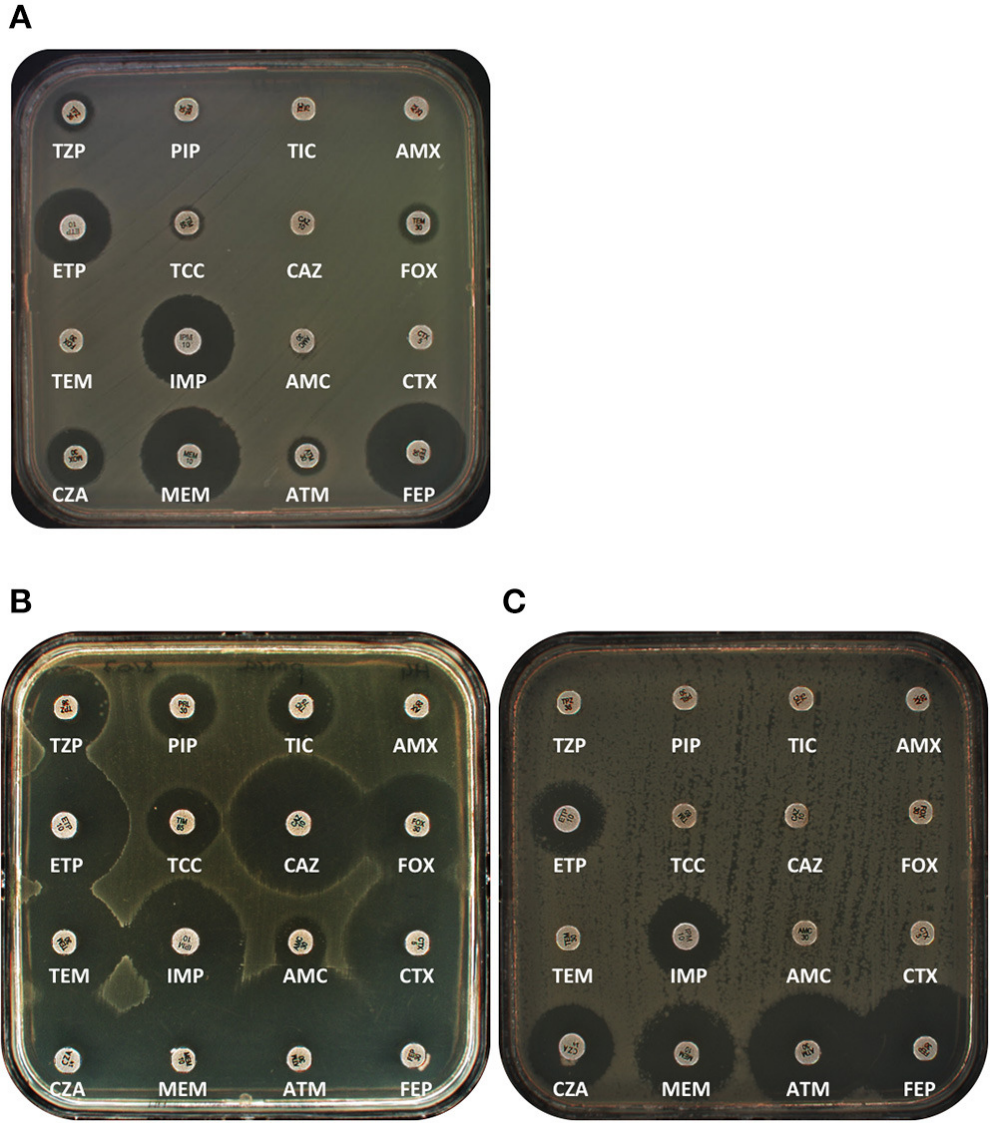
Antibiograms of representative class C & D carbapenemases. **(A)** ACT-28-producing *E. kobei* clinical isolate. **(B)** OXA-23 producing *P. mirabilis* clinical isolate. **(C)** OXA-372-producing *C. freundii* clinical isolates. AMX, Amoxicillin; AMC, Amoxicillin/clavulanate; ATM, aztreonam; CAZ, ceftazidime; CTX, Cefotaxime; CZA, Ceftazidime/avibactam; ETP, Ertapenem; FEP, Cefepime; FOX, Cefoxitine; IPM, Imipenem; MEM, Meropenem; PIP, Piperacillin; TCC, Ticarcillin/Clavulanate; TEM, Temocillin; TIC, Ticarcillin; TZP, Piperacillin/Tazobactam.

The other class C β-lactamase with potential carbapenemase activity is ACT-28 (110). ACT-1 was firstly identified in a carbapenem resistant *K. pneumoniae* in US (116). Lately, it has been demonstrated that the progenitor of *bla*_ACT−like_ genes was the *Enterobacter* genus (117). ACT-28 was firstly identified in eight carbapenem non-susceptible *Enterobacter kobei* sent to the French National Reference Centre for antimicrobial resistance (110). All isolates presented a positive carbapenem hydrolysis using the Carba-NP test. ACT-28-producing *E. kobei* isolates were resistant to penicillins, to penicillin/inhibitor combination, to broad-spectrum cephalosporins except cefepime, to aztreonam, but remained susceptible to carbapenem according EUCAST guidelines (Figure 3) (110). Purified ACT-28 exhibits low catalytic activity for imipenem (*k*_cat_ = 0.025 s^−1^) but presents a high affinity (*K*_m_ = 1.9 μM) resulting in a catalytic efficiency (*k*_cat_/ *K*_m_) at 0.013 μM^−1^.s^−1^, which is twice the value of ACT-1 purified in parallel. This small difference of catalytic efficiency might explain the positivity of detection tests based on imipenem hydrolysis. Furthermore, despite the fact that this catalytic efficiency is low, it is in the same range as other carbapenemases such as OXA-23 (Table 2). Regarding the genetic context, the *bla*_ACT−28_ was carried on the chromosome of a lineage of *E. kobei* ST-125. No mobile element was identified at the vicinity of this gene, indicating that this gene was not acquired but belongs to the core genome of this lineage. Search in the NCBI database identified this gene in different countries, including Brazil, USA, and UK, always in *E. kobei* of ST-125.

## Minor Class d Carbapenemases

In Enterobacterales, the main class D carbapenemase is OXA-48 that has spread worldwide with endemic area in Mediterranean countries with the exception of Israel, Greece, and Italy for which KPC-like enzymes represent the main carbapenemase. In this part of the manuscript, we will focus on the class D carbapenemases other than OXA-48-like enzymes. We can divide these enzymes in two groups: (i) carbapenemases firstly identified in other bacterial families before secondary spreading in Enterobacterales (OXA-23, OXA-40, OXA-58, & OXA-198) and (ii) carbapenemases identified firstly in Enterobacterales (OXA-372 & OXA-427).

### OXA-23

The *bla*_OXA−23_ gene codes for the main carbapenemase identified in *Acinetobacter* genus (118). In *Acinetobacter* spp. OXA-23 production confers high level resistance to all β-lactams. The *bla*_OXA−23_ gene was identified on many different conjugative plasmids, including GR6 incompatibility group, the most distributed plasmid family in *Acinetobacter baumannii* (119). The *bla*_OXA−23_ gene is most often found several transposons associated with IS*Aba1* (e.g., Tn*2006* or Tn*2008*) or IS*Aba4* (Tn*2007*) (120, 121). This cabapenemase has been reported worldwide but until 2002, it has never been described out of the *Acinetobacter* genus. The first report of OXA-23-producing Enterobacterales was published in 2002 with the description of 10 isolates recovered from 1996 to 1999 in a French hospital. Clonal relationship identified that all these isolates were clonally related despite they were recovered over a 4-year period without any link between patients (122). Since then, several report of OXA-23-producing *P. mirabilis* were reported in France, Singapore, and Finland (123–125). Intriguingly, the genomes of 20 OXA-23-producing *P. mirabilis* isolates recovered from France and Belgium have been compared to all *Proteus* genomes available in the NCBI database. This study highlighted that all isolates belonged to a single lineage that has spread since 1996 (126). Moreover, veterinary samples were included in this study and revealed that this lineage has spread also in animals (126). Analysis of the genetic context revealed that the *bla*_OXA−23_ gene is mainly carried on the chromosome of *P. mirabilis* (122, 124, 127) except for one isolate from France and three isolates from Singapore. There, the *bla*_OXA−23_ gene has been identified carried on an *Aba*R4-like structure on an untypeable plasmid (125). The *Aba*R4 structure is composed of Tn*2006*, carrying the *bla*_OXA−23_ gene, embedded in a complex transposon usually inserted in *comM* gene on the chromosome of *A. baumannii* (128). Regarding the other isolates, the *bla*_OXA−23_ is carried by complex IS*26*-based transposon. However, it is always associated to IS*Aba1* in its 5' extremity leading to its expression (126). OXA-23-producing *P. mirabilis* can be hard to detect. Indeed, MICs to carbapenems are relatively low and may be hidden by the low affinity of PLP for imipenem in *Proteus*. A moderate increase of carbapenem MICs can be observed from 0.25 to 0.5 mg/L but the presence of this carbapenemase should be evocated mainly in case of an increase of MICs for amoxicillin-clavulanate combination (Figure 3) (129). In addition to the difficulty to detect a phenotype associated to the production of OXA-23, most of carbapenemase detection tests for Enterobaterales do not detect carbapenem hydolyzing class D β-lactamases (CHDLs) other than OXA-48 (126).

### OXA-24/40

The *bla*_OXA−24/−40_ was firstly identified in *Acinetobacter baumannii* isolates from Spain and then described in several countries (130). Only one description of OXA-24-producing *P. mirabilis* is reported to date (131). No information is available regarding the genetic context of *bla*_OXA−24/−40_, except the impossibility to transfer the carbapenemase by conjugation.

### OXA-58

The last major CHDL frequently identified in *Acinetobacter* is OXA-58. This carbapenemase was firstly identified in France in 2003 (132) but then has been reported worldwide in *Acinetobacter* genus (118). Since recently, this carbapenemase was limited to *Acinetobacter spp*. The first report of OXA-58 in Enterobacterales was published in 2013 from Sierra Leone (133). However, no genetic is available with these isolates. Intriguingly, in addition to the *bla*_OXA−58_ gene, some *K. pneumoniae* possessed the *bla*_OXA−51_-like gene also, which is the natural β-lactamase of *A. baumannii*, suggesting a misidentification of the bacterial isolates or a contamination with *Acinetobacter* DNA. Since then, three recent studies regarding OXA-58-producing *P. mirabilis* from Belgium, Poland, and Germany were published (134–136). Genetic analysis revealed that isolates from Poland and Germany shared the same genetic context. The *bla*_OXA−58_ was carried by a small untypeable plasmid of 6.2 kb also carrying *aadA14* aminoglycoside resistance gene (135, 136). On the other hand, the Belgium isolate possessed a peculiar genetic context. The *bla*_OXA−58_ gene was localized on the chromosome associated to a class C β-lactamase *ampC* gene and was repeated in tandem (134). In all isolates, fragments of IS*Aba3*-like were identified at the vicinity of *bla*_OXA−58_ as observed in *Acinetobacter*. IS*Aba3* was described as the probable mobile element at the origin of the expression of *bla*_OXA−58_ in *Acinetobacter* (137). It is likely that the same promoter was conserved in *P. mirabilis* explaining the conserved fragments of IS*Aba3*.

### OXA-198

OXA-198 is a CHDL initially described in *P. aeruginosa* (138). After this sole occurrence, it was recently described in a *Citrobacter pasteurii* clinical isolate from France (139). In *P. aeruginosa, bla*_OXA−198_ is carried on an IncP11 plasmid of 49 kb (140), but in *C. pasteurii*, it was carried on an IncHI-type of 183 Kb (139). In both cases, *bla*_OXA−198_ is embedded in a class 1 integron. In *P. aeruginosa, bla*_OXA−198_ is the second cassette whereas in *C. freundii*, it corresponds to the third cassette (139). To date, this carbapenamase has been identified only in France and Belgium. This carbapenemase confers only a slight decrease of susceptibility to carbapenems (MICs of 0.38 mg/L, 0.5 mg/L, and 1 mg/L for meropenem, ertapenem, and imipenem, respectively in the *C. pasteurii* clinical isolate) (Table 1). In addition, this carbapenemase confers resistance to penicillins/inhibitor combination but spares ceftazidime. Catalytic constants confirmed this resistance phenotype with hydrolysis of penicillins and carbapenems but no activity toward broad-spectrum cephalosporins (Table 2).

### OXA-372

OXA-372 belongs to a new family of CHDLs described in Enterobacterales (141). This carbapenemase has been identified in *C. freundii* recovered from hospital wastewater in Italy (141). OXA-372 confers resistance to penicillins and diminished susceptibility to carbapenems but not to broad-spectrum cephalosporins, a common phenotype associated to the production of CHDLs (Figure 3; Table 1). Kinetic parameters confirmed that this enzyme is able to hydrolyze penicillins and carbapenems but not broad-spectrum cephalosprins and aztreonam (Table 2). Analysis of the genetic context revealed that the *bla*_OXA−372_ gene was carried by a multireplicon plasmid (IncA/C & IncN). This gene was embedded into a complex structure made of reminiscence of Tn*6017* itself inserted in Tn*6256*, a Tn*3*-family transposon.

### OXA-427

The last family of CHDL described in Enterobacterales corresponds to OXA-427 (142). This carbapenemase has been described in Belgium from various Enterobacterales being *K. pneumoniae, E. coli, K. oxytoca, S. marcescens*, and *Providencia rettgeri*. This carbapenemase confers resistance to penicillins including temocillin, ceftazidime, aztreonam, and ertapenem but spares cefotaxime (Table 1). Genetic analysis revealed that *bla*_OXA−427_ was localized on an IncA/C-type plasmid of 177kb (142). Immediate genetic context is made of a class 1 integron upstream of *bla*_OXA−427_ and a copy of IS*1326* dowstream (142). A recent study identified the *bla*_OXA−427_ on a multi-replicon plasmid IncA/C-InFIb-like plasmid (143). This plasmid of 321 kb actually resulted of the co-integration of the IncA/C-type plasmid carrying *bla*_OXA−427_ and an IncFIb-like plasmid (143). Biochemical analysis confirmed that OXA-427 is able to hydrolyze ceftazidime and imipenem (142), and was inhibited by avibactam (144).

## Detection Methods for Rare Carbapenemases

Since it is difficult (or impossible) to prevent the emergence of carbapenemases and more generally resistance genes, the most powerful method to bend their spread is to detect them. Indeed, earliest detection associated to hygiene measures and antimicrobial stewardship are our armamentarium against those bugs (145–147).

Mirroring the spread of carbapenemases numerous detection tools have emerged. These tests may be classified in different families based on their detection technology: (i) phenotypic tests, (ii) enzymatic tests based on hydrolysis, (iii) immuno-chromatographic assays, and (iv) molecular tests. This part of the manuscript will focus on the abilities of these tests to specifically detect rare carbapenemases and not on their global performance. Of note, taking in account the low number of isolates producing these rare cabapenemases, sensitivity and specificity might be false.

### Electrochemical Assay

Among the biochemical tests, the BYG test, named after the name of developers, is an electrochemical assays able to detect carbapenemase activity *via* a variation of conductivity during the carbapenem hydrolysis (148). During its multicenter evaluation, the BYG test was able to detect all GIM-1 (*n* = 1), FRI-1 (*n* = 1), SME-like (*n* = 2) producers. However, only 5/7 IMI/NMC-A producing isolates and no GES-5 producers (0/4) could be detected (148).

### Colorimetric Biochemical Assays

Different biochemical colorimetric tests based on the hydrolysis of carbapenem have been developed. The first test, the Carba-NP test, can detect imipenem hydrolysis *via* the production of acidic derivatives of imipenem (149). This test, along with the commercial test RAPIDEC® Carba NP (Biomérieux, France), has been extensively tested on major carbapenemases (149–151). Carba NP test was able to detect IMI/NMC-A-like, SME-like, FRI-1, and GIM-1 enzymes but failed to detect some GES-5 producing isolates (151). Another test based on colorimetric changes after hydrolysis of a chromogenic β-lactam is the β-CARBA™ test (Biorad, France) (152). This test showed similar sensitivity/specificity for the detection of the main carbapenemases compared to the Carba NP test and its commercial version, the RAPIDEC® Carba NP (152). It was also efficient in the detection of GIM-1. But it systematically failed to detect all minor class A carbapenemases including IMI-/NMC-A-like, SME-like, GES-5, and FRI-1 (151, 153). This result might be explained by the fact that the chromogenic β-lactam included in the β-CARBA™ test do not correspond to a true carbapenem, but a broad-spectrum cephalosporin that is likely not hydolzyzed by these minor class A carbapenemases.

### MALDI-TOF Based Detection of Carbapenem Hydrolysis

The last group of β-lactam hydrolysis-based detection assay correspond to the use of MALDI-TOF for the detection of a carbapenemase activity (154). Several detection tests using MALDI-TOF were developed including only one commercial kit, the MBT STAR® Carba IVD Kit (Brucker). These tests are based on the detection of (i) the disappearance of the carbapenem peak and (ii) the concomitant appearance of the peak corresponding of the hydrolyzed carbapenem after incubation of the carbapenemase-producing bacteria in a carbapenem supplemented solution. Overall, these tests showed good sensitivity/specificity for the detection of the main carbapenemases (154, 155). A multicentric evaluation of two methods, an in-house MALDI-TOF based protocol and the MBT STAR® Carba IVD Kit, demonstrated that the two methods were able to efficiently detect IMI-/NMC-A-like, SME-like, GES-5, GIM-1, and FRI-1 producing isolates (155).

### Hodge Test

One of the first phenotypic tests used for the detection of carbapenemase producers is the modified Hodge test, as known as cloverleaf test (156). Due to high number of false positive results as well as the weak sensitivity, this test is no longer considered as a good alternative for the detection of CPE including rare carbapenemase (156, 157). Of note, another modified Hodge test, Triton Hodge test, was developed by addition of Triton X-100 during the process (158). This test demonstrated good sensitivity to detect carbapenemases including NMC-A, SME-1, and GES-5 (produced by *P. aeruginosa*) (158).

### CIM Test

Another phenotypic test based on indirect detection of carbapenemase production corresponds to CIM test, for *C*arbapenem *I*nactivation *M*ethod, and derivatives (159). The aim of this test is to detect the ability of a carbapenem susceptible bacteria to grow close to a carbapenem containing disc after incubation of this disc with the suspected carbapenemase-producing bacteria. In a retrospective and prospective evaluation, this test revealed a good specificity and sensitivity for the detection of IMI-1/-2/-3, NMC-A, SME-1/-2, FRI-1, GIM-1, GES-5, and OXA-372 producers (160). The mCIM, *m*odified *C*arbapenem *I*nactivation *M*ethod, corresponds to a CIM test for which water was replaced with trypticase soy broth during the incubation phase between the carbapenem containing disc and the tested strain, and the time was extended (161). This test was able to detect the rare carbapenemases tested being NMC-A-like and SME-like enzymes (161). One of the most critical features with these tests is the 24 h delay to obtain the results. Another CIM derivative, rCIM for *r*apid *C*arbapenem *I*nactivation *M*ethod, was developed to target carbapenemase production in Enterobacterales (162). This test used a nephelometer to evaluate the growth of the susceptible bacteria instead of using a plate allowing a faster evaluation of the growth (few hours instead of 24 h). This tests accurately detected FRI-1, GES-5, IMI-1/-2, SME-1/-2, GIM-1, and OXA-372 (162).

### Inhibition Phenotypic Tests

Among the phenotypic detection tests, a wide diversity of combined disk methodologies has been developed. The combined disk assays are based on the use of carbapenem impregnated disk associated to different inhibitors such as boronic acid, dipicolinic acid, and cloxacillin, inhibiting class A, B, and C β-lactamases, respectively. As observed with the CIM and the mCIM, a 24 h delay is required to obtain results. Few studies tested the accuracy of this test on rare carbapenemases. Recently, the Carbapenemase Detection Set® (MAST Diagnostic) was evaluated (163). This assay accurately detected IMI-/NMC-A-like, SME-1/-2, and FRI-1 as class A carbapenemase, GIM-1 as a class B, but complementary tests were needed to decipher the presence of GES-5 (due to the absence of any diameter differences with all tested inhibitors) (164). Other combined disc methods are commercially available but were not evaluated on rare carbapenemase-producing Enterobacterales.

### Lateral Flow Immunoassays

Recently, lateral flow immunoassays have been developed for the detection of the main carbapenemases. The main tests are RESIST-4 O.K.V.M (Coris Bioconcept), which detect KPC, NDM, VIM, OXA-48-like enzymes, and NG-Test® CARBA 5 (NG Biotech), which detects KPC, NDM, VIM, OXA-48-like, and IMP enzymes. Both of these tests can deliver results in <15 min when performed on bacterial colonies (165–167) or directly from positive blood cultures (168, 169). Despite both of these tests possess excellent performance for the detection of the “Big five” carbapenemases encountered in Enterobacterales, none of the rare carbapenemase are included in the detection panel yet. More recently, the OXA-23 K-Set® (Coris Bioconcept) has been developed for the detection of OXA-23-producing *Acinetobacter* spp. However, OXA-23 is also rarely identified in *P. mirabilis*. Accordingly, a recent evaluation demonstrated that OXA-23 K-Set® accurately detects OXA-23-producing *P. mirabilis* (170).

### Molecular Detection of Carbapenemase Encoding Genes

The last group of detection tests gathers the molecular test in which PCR is the warhorse for the detection of carbapenemase encoding genes. By contrast to biochemical tests that detect carbapenemase activity, molecular tests detect the presence of a specific gene. Thus, the main caution of these tests is “*we are able to detect only what we target*.” One of the most worldwide spread molecular assay for the detection of carbapenemase encoding genes corresponds to the GeneXpert® (Cepheid) (171). The current version Carba-R V2 is able to detect accurately and “Big Five” carbapenemase encoding genes but none of the rare carbapenemase encoding ones (172). Currently, this issue is common for most of PCR-based detection kit including Revogene (Meridian bioscience), Biofire filmArray Blood Culture identification panel (BioMérieux) Amplidiag CarbaR+MCR (Mobidiag), Luminex xTAG assay (Luminex corp), Check-MDR CT103 (Check-Points Health), or CRE ELITe MGB® (Elitech) kits (173–177). Interestingly, the *bla*_OXA−23_ and *bla*_OXA−58_ genes are detected by Amplidiag CarbaR+MCR kit.

Whole genome sequencing, despite not based directly on PCR, is a molecular method very useful for precise identification of resistance mechanism to carbapenems. The main weakness of this technology is that a genotype may not explain a phenotype and vice versa. Indeed, the presence of a gene does not necessarily prove its expression. That is the reason why *in silico* antibiogram is not widely used yet (178). However, this method is able to detect any gene related to β-lactamases whatever its homology or phenotype as well as undetected or totally novel β-lactamase family. Moreover, servers for analysis of WGS raw data are not appropriate for an easy interpretation of the data, e.g., Resfinder (179), CARD (180).

## β-Lactamase Inhibitors and Rare Carbapenemases

The use of β-lactamase inhibitors is crucial to fight β-lactamase-producing isolates. Among the well-known inhibitors, the three inhibitors of class A β-lactamases, clavulanate, tazobactam, and sulbactam, are widely used in clinical practice (181). Unfortunately, carbapenem-resistant Enterobacterales (and particularly carbapenemase-producing Enterobacterales) are most often resistant to the classical β-lactam/β-lactamase inhibitors associations (e.g., amoxiciline-clavulanate, ticarcilline-clavulanate, piperacilline-tazobactam, ceftolozane-tazobactam) used to treat infected patients. Thus, an urgent need for new inhibitors was obvious. Recently, several inhibitors have been developed. Among them three inhibitors are now approved or in phase 3, avibactam (belonging to diazibyciclooctanone DBO), relebactam (DBO), and vaborbactam (boronic acid derivative) (182). Avibactam, formerly NXL-104, in combination with ceftazidime, proved its efficacy against class A, C, and some class D but not against class B β-lactamases (183). Among rare carbapenemases, avibactam demonstrated efficacy against CTX-M-33-, GES-5-, SME-2-producing isolates (42, 184). To this short list, zidebactam, a new DBO member, can be added (185), nacubactam, a bridged DBO, enmetazobactam, belonging to penicillanic acid sulfone class, taniborbactam a boronic acid derivative (186–188). Avibactam inhibits class A, C, and D whereas relebactam and vaborbactam mainly inhibit class A and C (189). Activity of vaborbactam in combination with meropenem has been described toward some rare class A carbapenemase such as SME-, NMC-A-, FRI-1-, and BKC-1-producing isolates (190). However, this combination has limited activity against class B (NDM-, VIM-, or IMP-producing isolates) and class D (OXA-48-like-producing isolates) (191). Relebactam demonstrated inhibition toward KPC-, SHV-, CTX-M-,TEM-, or class C-β-lactamases but exhibited moderate inhibition against OXA-48-like (183). However, it has been observed that, despite activity against class A carbapenemases such as KPC, relebactam does not inhibit SME-4 enzyme (192). A GES-20-producing *K. pneumoniae* resistant to imipenem-relebactam was also reported (193). Cefepime-enmetaozactam, formerly AAI101, demonstrated activity against ESBLs- or AMPc-producing isolates but limited activity toward KPC- and VIM-producing isolates (183). No data regarding its activity against minor carbapenemase is available. Zidebactam (formerly WCK 5107) exhibited activity against class A and B carbapenemases and moderate inhibition activity OXA-23/-40/-58-producing *A. baumannii* (194). Noticeably, zidebactam also inhibits PBP2 and thus possess intrinsic antibacterial activity (183). Among minor carbapenemases, cefepime/zidebactam demonstrated activity against a GES-18-producing isolate (194). Nacubactam, formerly FPI-1465, demonstrated *in vitro* inihibition against class A, C, and some class D β-lactamases (183). As observed for zidebactam, Nacubactam demonstrated affinity to PBP2 and thus also exhibited activity toward MBL-producing isolates (188). This molecule remains to be tested for minor carbapenemases. Taniborbactam, formerly VNRX-5133, is able to inhibit class A, C, and D β-lactamases and even class B carbapenemases (187, 195). Among MBLs inhibited by VIM-, NDM-, SPM-1-producing isolates but not IMP-like enzyme (187). Of note, GIM-1 and GES-5 are inhibited by taniborbactam (195).

## Conclusions

Since the first description of “an enzyme able to destroy penicillin,” thousands of β-lactamases were identified from more than 50 families. The wide genetic diversity associated to very diverse phenotypes largely complicate the identification of the resistance mechanisms involved in carbapenem resistant Enterobacterales. Although several tools have been developed for the accurate detection of the 5 main carbapenemases, KPC, NDM, VIM, IMP, and OXA-48-like, it might be now interesting to developed multiplex tests (molecular tests or immunochromatographic assays) that will be able to fill the gap in the detection of rare carbapenemase-encoding Enterobacterales. It might be of particular interest for Ambler class A carbapenemases of GES-, IMI-, SME-, and FRI-type that have already been reported in different countries, and for which some of the widely used colorimetric biochemical tests (e.g., β-CARBA™ test) remain inefficient. Without efficient detection tools, these enzymes might be a concern in a near future in healthcare facilities.

## Author Contributions

All authors listed have made a substantial, direct and intellectual contribution to the work, and approved it for publication.

## Conflict of Interest

The authors declare that the research was conducted in the absence of any commercial or financial relationships that could be construed as a potential conflict of interest.
